# A Viral Discovery Methodology for Clinical Biopsy Samples Utilising Massively Parallel Next Generation Sequencing

**DOI:** 10.1371/journal.pone.0028879

**Published:** 2011-12-21

**Authors:** Gordon M. Daly, Nick Bexfield, Judith Heaney, Sam Stubbs, Antonia P. Mayer, Anne Palser, Paul Kellam, Nizar Drou, Mario Caccamo, Laurence Tiley, Graeme J. M. Alexander, William Bernal, Jonathan L. Heeney

**Affiliations:** 1 Department of Veterinary Medicine, The University of Cambridge, Cambridge, United Kingdom; 2 Wellcome Trust Sanger Institute, Wellcome Trust Genome Campus, Hinxton, Cambridge, United Kingdom; 3 The Genome Analysis Centre, Norwich Research Park, Norwich, United Kingdom; 4 Department of Hepatology, Addenbrookes Hospital, Cambridge University Hospitals National Health Services Foundation Trust, Cambridge, United Kingdom; 5 Institute of Liver Studies, King's College London School of Medicine, King's College Hospital, Denmark Hill, London, United Kingdom; University of Texas Medical Branch, United States of America

## Abstract

Here we describe a virus discovery protocol for a range of different virus genera, that can be applied to biopsy-sized tissue samples. Our viral enrichment procedure, validated using canine and human liver samples, significantly improves viral read copy number and increases the length of viral contigs that can be generated by *de novo* assembly. This in turn enables the Illumina next generation sequencing (NGS) platform to be used as an effective tool for viral discovery from tissue samples.

## Introduction

A variety of methods for identifying unknown viruses have been reported, such as: degenerate primer PCR/amplification [1], viral microarrays [2]–[4] and conventional sequencing. Low abundance of viral sequences relative to total host nucleic acids usually requires the use of viral enrichment and concentration procedures. These include: filtration, ultracentrifugation and nuclease treatment followed by random priming and amplification using the sequence-independent single primer amplification (SISPA) method or variations thereof [1], [5]–[10] and/or with the Viral Discovery cDNA-AFLP (VIDISCA) method [11]–[15]. These approaches have generally been limited to liquid based samples (body fluids, eluted swabs, culture supernatants and environmental samples). NGS has shown great potential for novel virus discovery [16]–[19]. The use of NGS alone can be sufficient if the viral nucleic acids are in sufficient abundance relative to host nucleic acids. However, as we confirm here, clinical biopsy samples can present a problem where even the depth of sequencing provided by NGS may be insufficient to generate useful viral sequence contigs by *de novo* assembly.

We have now established a broadly applicable approach for viral nucleic acid enrichment from small biopsy sized clinical liver tissue (e.g Tru-Cut), which combined with the Illumina NGS platform could provide an effective tool for viral discovery.

## Results

### Detection of HCV reads from HCV infected human biopsy samples using the Illumina platform

**Figure 1:**
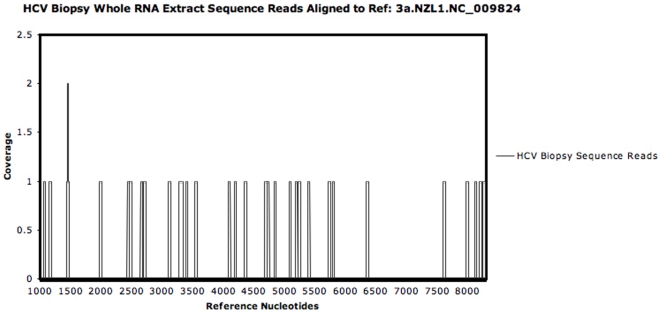
NGS of HCV biopsy RNA. Extracted and pooled (×6) HCV infected biopsy RNA was reverse transcribed and amplified prior to Illumina NGS and mapped to the HCV reference 3a.NZL. NC_0009824 (Los Alamos HCV database).

We analysed frozen Hepatitis C virus (HCV) infected Tru-Cut liver biopsies without viral enrichment to ascertain the limitations of detection of virus in a small liver biopsy using the Roche 454 and the Illumina NGS platforms. Total RNA was extracted from six biopsy samples (RNA integrity (RIN) values between 6 and 8) and HCV infection was confirmed by PCR. 0.5 µg of RNA from each sample were pooled and underwent SISPA (detailed in materials and methods). The minimally amplified pooled material was then mass sequenced on a single Illumina NGS lane. Mapping of the short reads to HCV reference genomes from the Los Alamos HCV database confirmed HCV infection with sub-type 3a. However, the mapping clearly showed a paucity of viral genome-coverage (12.5%) with a total of 32 HCV reads out of ∼8 million (Fig. 1). tBLASTx analysis of the complete dataset of viral fragments against all HCV genomes in the EMBL database did not identify any further HCV reads. The lack of overlapping viral sequence reads prevented *de novo* assembly of viral contigs, making the use of the Illumina NGS platform and the SISPA protocol alone a potentially ineffective technique for novel virus discovery. The same process using the Roche 454 platform failed to identify any HCV sequences.

### Liver Cytosol/Pellet fractionation for viral enrichment

**Figure 2:**
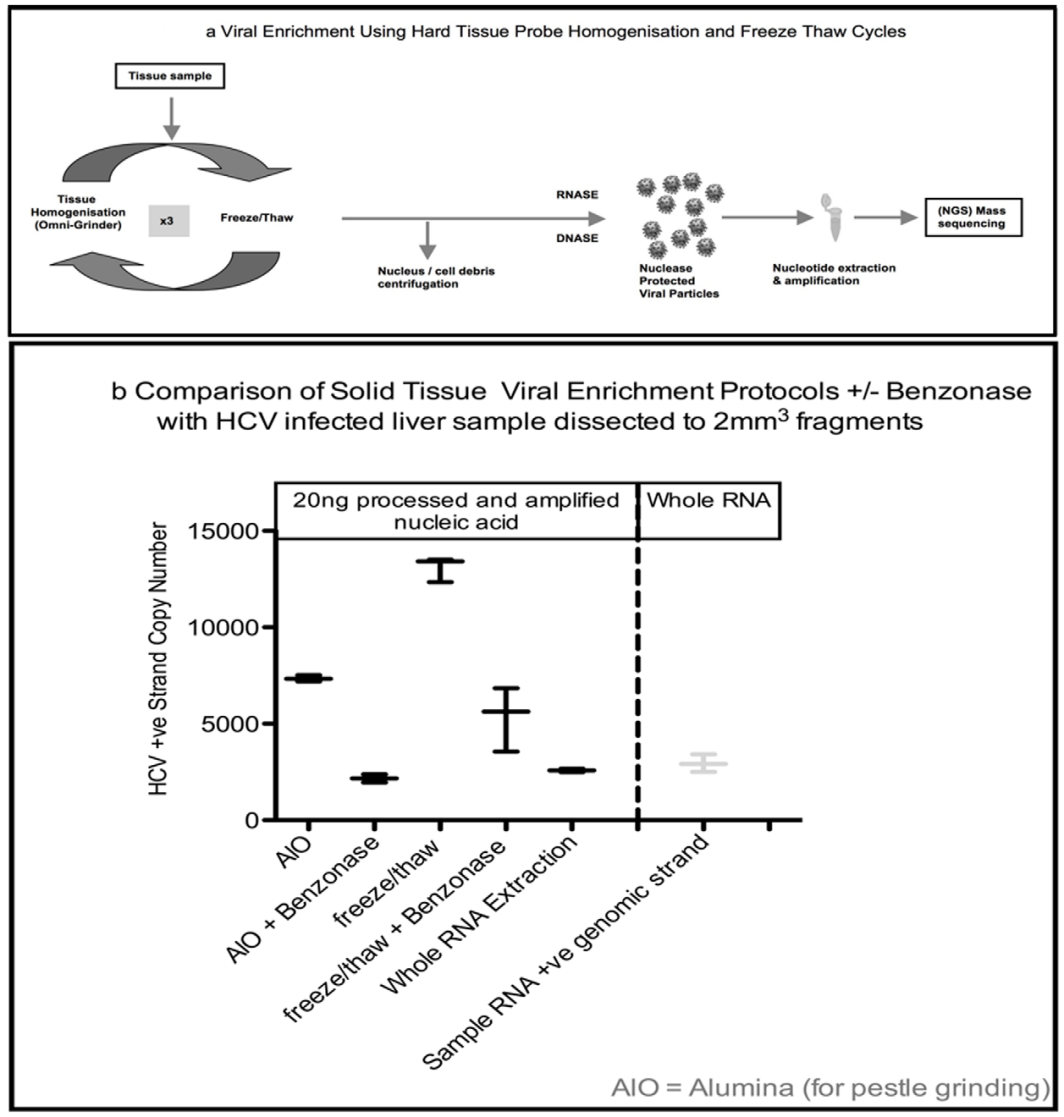
Enrichment methodologies. **a:** Illustration of the key steps in isolating and enriching viral nucleic acids relative to host nucleic acids prior to sequencing by NGS. Liver tissue cells were broken in an isotonic buffer supplemented with BSA using a hard tissue probe and an Omni-Homogeniser with a dry-ice freeze/thaw step (repeated 3×). To ensure that cells membranes were broken the lysate was checked microscopically. **b:** Comparison of Alumina/pestle-grinding against hard-tissue probe homogenisation with freeze/thaw cycles (+/− the addition of Benzonase). (2 mm^3^ fragments were dissected from an HCV infected liver sample. Duplicate tissue samples were used for each protocol. HCV nucleic acids were measured in triplicate by dual labelled qPCR assay with NIBSC standard.

To improve on published tissue extraction methods for viral discovery [20] we compared different homogenization procedures. We found the optimal procedure was the use of a TH Omni-homogeniser/hard-tissue probe combination (Omni-International) using a short pulse (15 seconds) in cold PBS with a dry ice freeze thaw cycle, repeated three times, followed by RNAse and DNAse digestion of the host nucleic acids as illustrated in figure 2a and detailed in materials and methods. We found that pestle grinding with Alumina did not break the liver cells as reliably as our freeze/thaw-mechanical approach (determined microscopically).

We compared the two methods with dissected (2 mm^3^) biopsy sized fragments of an HCV infected liver sample with and without the presence of Benzonase as a nuclease to remove host nucleic acids (Fig. 2b). Following nuclease treatment with both Turbo-DNAse and RNAse1 (+/− Benzonase) to remove host nucleic acids, we extracted viral genomic material and residual host nucleic acids using the Trizol RNA extraction method with glycogen as a carrier. A modified SISPA protocol (materials and methods) was used to amplify a minimum amount of material needed for the Illumina NGS platform (1–3 µg) after cleaning and size fractionation to remove sub-200 bp fragments. By removing the sub-200 bp fragments we were effectively comparing NGS readable nucleic acids whilst removing fragments likely to represent residual host material that had survived exposure to the nuclease treatment. We determined that the improved cell breakage (determined microscopically) with the probe homogenization and freeze thaw cycles, correlated with an improved concentration of HCV nucleic acids using identical amounts of post-amplification dsDNA as an input to an HCV qPCR assay. Furthermore, with both methods the addition of Benzonase together with RNAse1 and Turbo DNAse reduced the HCV copy number 2–6 fold.

**Figure 3:**
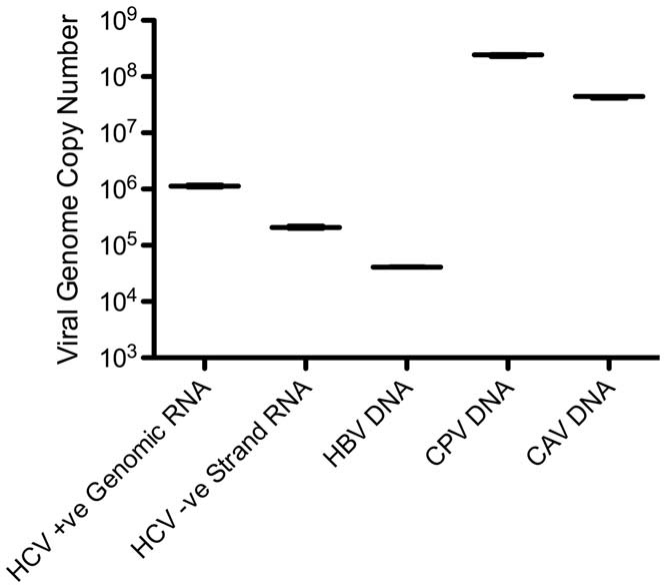
Quantitative PCR (viral genome copy number in liver samples). A SYBR-green assay and plasmid standard was used for the CAV and CPV samples, whilst the HCV and HBV assays used dual labelled probed with NIBSC standards. Total RNA extracted for HCV and Total DNA extracted for HBV, CPV and CAV. The Genomic strand copy number for HCV was estimated by performing the RT step in the presence of the reverse primer only.

To validate our extraction/enrichment approach (high-speed tissue homogenization and freeze/thaw cycles) with Illumina NGS, we used four pieces of liver tissue infected with canine parvovirus 2 (CPV2), canine adenovirus 1 (CAV1), human hepatitis B virus (HBV) and hepatitis C virus (HCV). Liver samples were dissected into 2 mm^3^ pieces (equivalent to approximately half of a Tru-Cut needle biopsy sample). Estimates of the viral sequence copy number per liver sample (2 mm^3^) were made by qPCR (Fig. 3) to confirm the presence of the viral nucleic acids within the liver tissue and to estimate the maximum possible starting viral copy number of the sample with the subsequent processed fractions.

**Figure 4:**
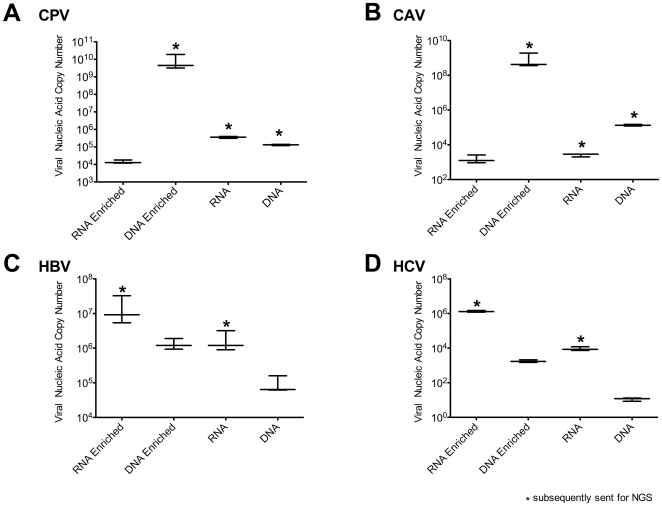
Quantitative PCR (viral copy number estimations of prepared samples for NGS). For each viral liver sample, 2 mm^3^ of liver was used separately for total RNA extraction, total DNA extraction, DNA viral enrichment and RNA viral enrichment. qPCR assays were performed using 20 ng of each sample (estimated by Pico-green assay) as an input in triplicate. The samples subsequently mass sequenced are indicated with an asterisk.

Next, our enrichment procedure and the total RNA and DNA extractions were carried out on separate 2 mm^3^ dissected fragments of each liver sample (materials and methods). Following nuclease treatment with turbo-DNAse and RNAse1 (without Benzonase) the SISPA based protocol was used to amplify a minimum amount of material needed for the Illumina NGS platform (1–3 µg) after cleaning and size fractionation to remove sub-200 bp fragments. The level of enrichment achieved, per nanogram of amplified material, for all four different virus infected liver samples was quantified by qPCR (Fig. 4). The DNA virus enrichment achieved a 10^4^ increase in CPV viral nucleic acids relative to the highest non-enriched CPV sample extract (total RNA) and a similar enrichment level for CAV relative to the highest non-enriched CAV sample extract (total DNA). The RNA virus enrichment process achieved a 10× increase in both HBV and HCV nucleic acids relative to the highest non-enriched samples (total RNA).

### Illumina NGS viral read and de novo assembly comparison

**Figure 5:**
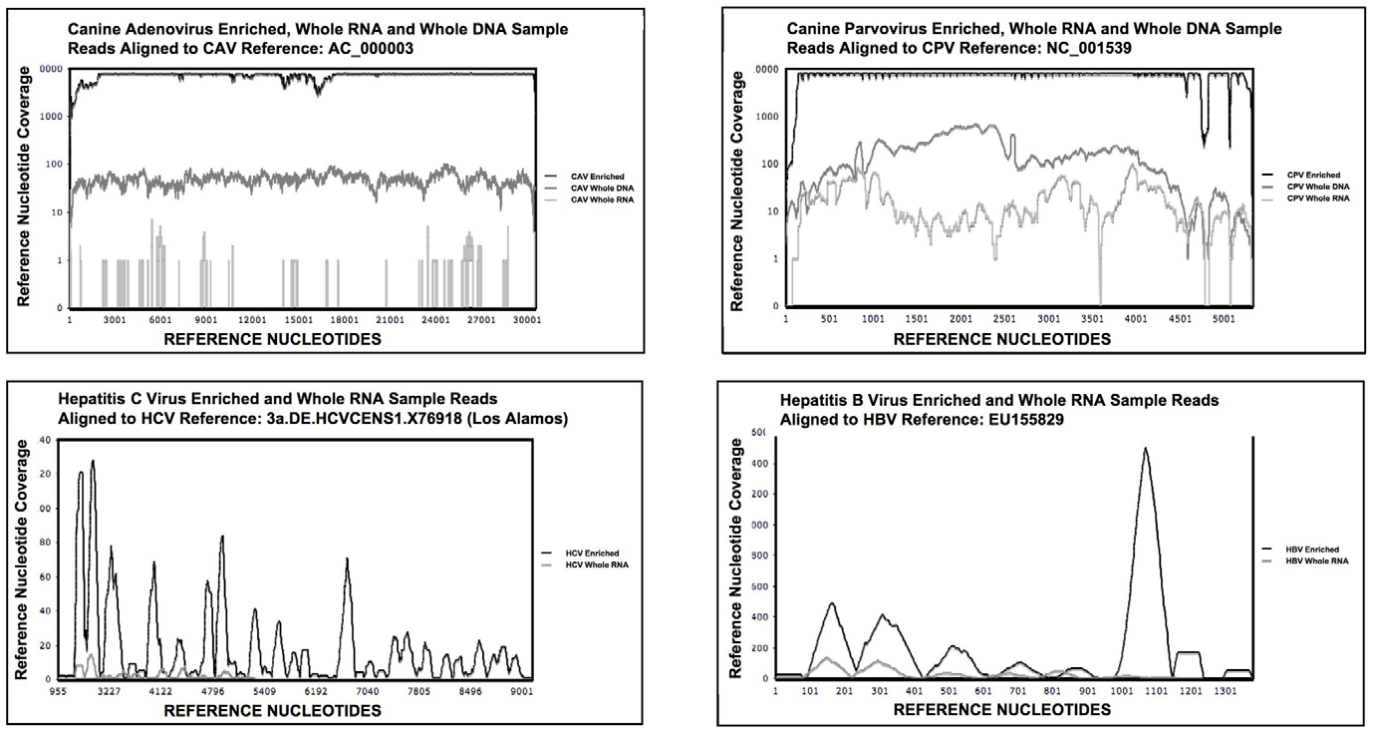
NGS virus sequence mapping to reference genomes. To assess the relative difference between total RNA and total DNA extracts with the virally enriched samples, the NGS viral reads were mapped to respective reference genomes.

**Figure 6:**
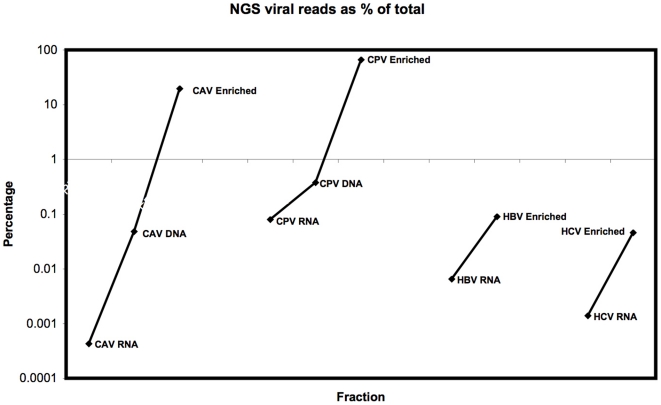
Virus reference reads as percentage of NGS datasets. Viral reference sequencing reads shown as a percentage of total total reads from the Illumina NGS datasets for the differentially processed liver samples.

Based on the qPCR results, ten samples were selected for mass sequencing on the Illumina NGS platform with one sample library per lane, indicated by an asterisk in Fig. 4. Mapping sequence reads (Fig. 5) revealed complete or near complete (>95%) coverage of the reference genomes for the virally enriched samples. Viral nucleic acid point-coverage was orders of magnitude (4×10^1^–8.5×10^4^) greater than total RNA (non-enriched) samples processed in the same manner. The increase in viral sequences in enriched samples as a percentage of the total NGS read set is shown in Fig. 6. Mass-sequencing of total DNA from CAV and CPV infected samples was performed since qPCR data indicated these samples had a higher viral genome copy number than the RNA transcriptome samples (Fig. 4). This correlated with the NGS read mapping analysis (Figs. 5 & 6) for both the CPV and CAV total DNA samples.

**Figure 7:**
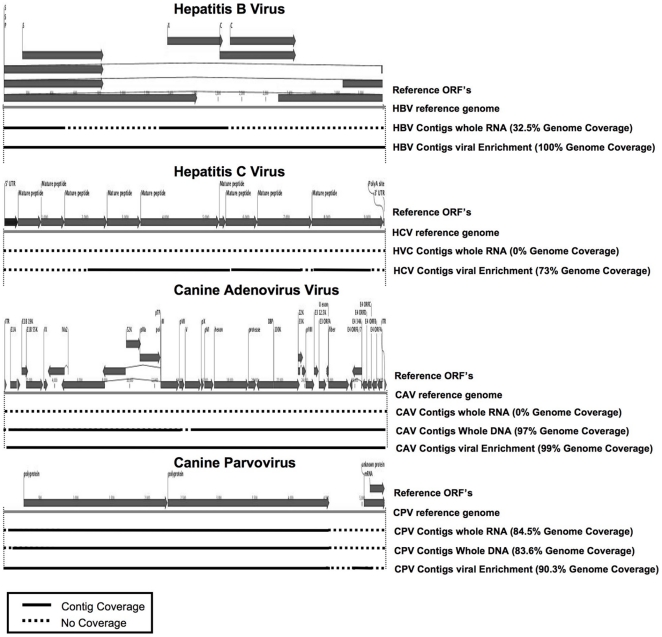
*De novo* assembly of viral contigs. NGS reads for infected liver samples and their processed fractions, were used to generate contigs using ABySS and CLC *de novo* assembly algorithms. Contigs (length over 200 nt) were mapped to the viral reference genomes with the length and coverage indicated (solid black lines). Regions of the reference genomes not covered by the contigs are indicated with dashed lines.

In order to ascertain whether the increased viral read copy number and reference coverage yielded significant improvement in the size and viral reference coverage of the contigs that could be generated by *de novo* assembly, we utilised CLC Genomics v4 (Katrinebjerg, 8200 Aarhus N, Denmark) and ABySS [21] *de novo* assemblers in tandem and with varying stringencies. The data from the unenriched CAV and HCV infected total RNA samples indicated that RNA extraction and SISPA processing alone was insufficient to generate contigs with significant viral reference coverage. The HBV infected RNA fraction produced two contigs covering one third of the HBV genome. In contrast, the virally enriched samples produced contigs covering between 73–100% of their reference genome (Fig. 7), with the HBV enriched preparation producing a single contig spanning 100% of the genome. The canine parvovirus was clearly transcriptionally active and the contig coverage from the total RNA (non-enriched) fraction was comparable to the virally enriched sample. For the CAV and CPV total DNA extracts, the resulting assembled contigs (1–2/sample) covered the reference genomes between 83.6% and 97% respectively, lower than, but comparable to the enriched viral DNA samples.

## Discussion

Identifying unknown viruses in clinical samples is technically challenging and this is especially true for solid tissues when compared with using less complex samples such as body fluids. Additionally, whilst biopsy sampling is routine in the clinic it can be limited for research purposes by ethical and safety considerations. Often, the amount of material available for virological analysis may be limited to a fraction of a small needle biopsy left-over after diagnostic histopathological analysis. Detection of virus is not always possible in serum and plasma even with nucleic acid tests whilst the ‘occult’ virus may still be detectable in the viral reservoir tissue [22]–[23].

Mass sequencing technologies provide a new avenue for viral discovery as highlighted by Feng and Palacios [17]–[18] using the Roche 454 NGS platform. The Illumina NGS platform also has scope for novel viral discovery, particularly with recent technical developments yielding improved sequence length and quality now complementing its exceptional sequencing depth. However, our preliminary work (Fig. 1) demonstrated that virally infected clinical biopsy samples exist where extracted total nucleic acids processed for NGS with the Illumina platform contain viral sequence at such a low number relative to host nucleic acids that viral reference coverage was sparse with no overlapping reads, precluding the possibility of assembling larger contiguous viral sequences. This is key in facilitating the discovery and characterization of novel viruses by nucleotide and amino acid similarity to viral database sequences or by predicted structural domain conservation. Concurrent NGS with the Roche 454 platform failed to sequence any viral nucleic acids that we could detect and for both platforms the only assembled contigs over 200 bp corresponded to host sequences.

We have shown that our enrichment methodology together with the Illumina NGS platform works for a range of viruses using sample sizes equivalent to a small needle biopsy. Cells in the sample are disrupted and the cytosol isolated with encapsidated virus intact. The resulting cytosolic fraction can be separated from the cell debris and nuclei leaving a low viscosity solution that can be nuclease treated directly or readily size fractionated by filtration methods. qPCR analysis of equivalent samples processed for DNA, RNA and with the viral enrichment method clearly demonstrate the extent of enrichment (Fig. 4) as did mapping of the subsequent NGS reads to the respective viral genomes to assess coverage (Fig. 5) and target viral sequence as a percentage of the whole NGS read set (Fig. 6). Subsequent *de novo* assembly of the NGS reads using different assembly algorithms (fig. 7) demonstrates the advantage of the enrichment procedure over whole RNA transcriptome sequencing. Enriched sample *de novo* assembled contigs using two different algorithms resulted in 73–100% coverage of the viral reference genome with one to three contigs. For the un-enriched RNA samples, only the CPV and the HBV sample contigs included contigs to the reference virus (97% and 32.5% respectively). Interestingly, it is clear that viral DNA genomes, from the CAV and CPV samples we used for validation, could be reconstituted from total extracted DNA using NGS alone. This is of particular interest if one considers that an unknown (DNA) virus may be latent, in a non-encapsidated, non-replicative and transcriptionally repressed form at a given time point or cell type. This is of course a hypothetical, but in such a case, total RNA and viral enrichment analysis would be unlikely to work. Furthermore, with this methodology, the DNA can be concurrently extracted from the nuclei containing pellet after homogenization and prior to nuclease treatment, thus a three way strategy might be considered when attempting to find an unknown virus in a solid tissue biopsy. Our technique readily allows total RNA, virus enrichable cytosolic fraction and total DNA to be extracted with minimal loss of sample and minimal sample size.

In summary, our method can enrich a range of virus types that can be sequenced using the Illumina NGS platform. Furthermore, viral genomes can be largely reconstituted by currently available *de novo* assembly algorithms. This approach is robust, enabling the use of NGS for the detection and identification of novel viral pathogens from small diagnostic biopsy samples without the requirement to culture or isolate the virus first. We show this technique works for liver tissue, which can be a difficult tissue for extracting high quality RNA from and the HCV sample we used for enrichment validation and NGS was approximately 8 mm^3^ of very hard fibrotic tissue that had a low viral genome copy number (Fig. 3) suggesting that the technique could probably be applied successfully to most tissue types including other fibrotic diseased tissues or joint collagen for example. Importantly, this methodology is rapid and results in very little loss of sample.

## Methods

### Liver samples and processing

Human liver samples were acquired from the Institute of Liver Studies, Kings College Hospital, London, University of London, UK. Samples were obtained with patient written consent. This work forms part of a broader project with ethical approval provided by the UK National Research Ethics Service, Cambridge 3 Research Ethics Committee, Cambridge CB21 5XB (REC reference numbers 09/HO306/52, 09/HO306/60) and Kings College Hospital Research Ethics Committee, London SE5 9RS (REC reference number 04/Q0703/27).

Canine liver samples were acquired from the Blue Cross Animal Hospital (CPV) and the Royal Veterinary College (CAV) with informed and written owner consent acquired by both centers as per the guidelines of the Royal College of Veterinary Surgeons (RCVS), UK. The CAV and CPV liver samples represented legacy material for which no ethical committee approval was required in the UK.

Individual samples were screened +ve for the following viruses (Table. 1). After collection, liver tissue was stored at −80°C pending further analysis. Liver samples were divided into sections measuring 2 mm^3^ and weighing ∼15 mg comparable to approximately half a Tru-Cut needle biopsy. Size rather than weight was used for handling reasons in order to keep the samples as cold as possible to minimize nucleic acid degradation.

**Table 1:** Characteristics of the different viral genera present in the liver tissue used in this study.

Virus	Group	Envelope +/−	Genome	Size (Kb)
**CAV-1**	**I**	**−**	**dsDNA**	**30.5**
**CPV-2**	**II**	**−**	**ssDNA −/+**	**5**
**HCV-3**	**IV**	**+**	**ssRNA +**	**10.5**
**HBV**	**VII**	**+**	**(Partial) dsDNA**	**3.1**

#### No Enrichment

Total RNA extraction was performed on liver tissue (Liver biopsy or 2 mm^3^) using the RNeasy Lipid Tissue Mini Kit (Qiagen) according to the manufacturer's instructions with on column DNAse treatment. Total DNA extraction was performed on liver tissue (2 mm^3^ or from nuclear pellet) using the QIAamp DNA Mini Kit (Qiagen) and according to the manufacturer's instructions. Liver tissue was homogenized in the relevant denaturant, according to the kit manufacturers instructions, using a micropestle (Eppendorf).

#### Enrichment

Liver tissue (2 mm^3^) was immersed in 250 µl ice cold 0.7% bovine albumen supplemented buffered saline pH.7.2 and homogenised for 15 seconds on ice using an Omni TH - Tissue Homogenizer and disposable (7 mm×110 mm) ‘Omni Tip’ hard-tissue probe (Omni-International). The resulting homogenate was placed on dry ice for approximately two minutes until frozen, and thawed quickly before returning to ice. Homogenization followed by freezing and thawing was repeated a further two times to disrupt the cells (>90%) while leaving the nucleus intact (>90%) determined microscopically. Samples were then spun at 600×***g*** for 10 minutes at 4°C to pellet the nuclei and large cellular aggregate. Non-particle protected viral DNA and RNA was removed from the supernatant by digestion with 30 U of turbo DNase [Ambion] and 25 U of RNase One [Promega]) in 1× DNAse buffer (Ambion) and incubated at 37°C for 90 minutes. Viral DNA (virally enriched) was extracted using the High Pure Viral Nucleic Acid Kit (Roche) according to the manufacturer's instructions and eluted with 30 µl water. The DNA virus extraction method used a polyA carrier not suitable for the viral RNA extraction with the necessity for subsequent random priming and amplification. We further established that glycogen was not an efficient substitute for polynucleotides as a carrier in silica column based methods. Therefore, viral RNA from the cytosolic extract was extracted using Trizol LS (Invitrogen) according to the manufacturer's instructions with modifications. 20 µg glycogen was added prior to precipitation and left over-night at −20°C and vortexed after 1 hour and 24 hours. The precipitation was then frozen at −80°C and vortexed again prior to centrifugation at 10 K*g* for 30 minutes at 4°C. The RNA pellet was rinsed three times in 75% ethanol, dried at room temperature and the pellet re-suspended in 20 µl water supplemented with 80 U RNAse OUT (Invitrogen). RNA was passed through a NucleoSpin RNA Clean-up XS column (Machery-Nagel) according to the manufacturer's instructions and eluted with 10 µl water supplemented with 80 U RNAse OUT.

Alumina grinding and the addition of Benzonase was used in a comparison study with a HCV infected liver sample using a previously reported protocol [20]. This was adapted for use with a needle biopsy sized tissue sample, obviating the need for ultracentrifugation to pellet the virus. Briefly, the liver tissue 2 mm^3^ was ground with a micropestle (Eppendorf) with 20 mg of 100-mesh Alumina (Sigma-Aldrich) and 250 µl of ice cold 0.7% bovine albumen supplemented buffered saline pH.7.2. The homogenized sample was spun at 2.5 k rpm for 20 minutes at 4°C and passed through a 0.45 micron low bind filter (Millipore). This was compared to an identical sized sample from the same specimen processed for enrichment using our freeze/thaw protocol described. Additionally, Benzonase was tested as an additional nuclease to remove host nucleic acids, with both processed samples using 20 U benzonase (Novagen).

### Sequence independent Single Primer Amplification (SISPA): RT/2^nd^ strand synthesis and product amplification

The protocol was adapted from previously reported SISPA based protocols^10,21–22^ and using the SISPA primers/adaptors:

FR26RV-N GCCGGAGCTCTGCAGATATCNNNNNN

FR20RV GCCGGAGCTCTGCAGATATC.

First-strand cDNA synthesis was performed using 100 U of Superscript III and the primer FR26RV-N (20 pmoles) using the manufacturers recommended random primer protocol (Invitrogen, UK) at 50°C for 60 minutes in the presence of RNase- OUT (Invitrogen, UK). cDNA and DNA were incubated with 2.5 U of Klenow DNA polymerase (NEB) at 37°C for 60 minutes with an inactivation step of 75°C for 20 minutes. PCR of the above extension products was performed using 5 µl of cDNA or DNA in a total reaction volume of 50 µl containing 2.5 mM MgCl_2_, 0.2 mM dNTPs, 1× Advantage 2 kit PCR buffer (Takara-Clontech), 0.8 mM primer FR20RV and 1 U Advantage 2 kit Polymerase Mix. Temperature cycling was performed as follows: 1 cycle of 95°C for 2 minutes, 20 cycles (minimum) of denaturing at 95°C for 30 seconds, 65°C for 1 minute, 68°C for 30 seconds. An additional extension of 3 minutes at 68°C was performed. Further cycling was used when necessary to generate a final output of 1–3 µg of dsDNA post-fractionation and clean-up. Amplified DNA/cDNA was cleaned and fractionated using Chroma-Spin-200 columns (Takara-Clontech) to remove sub-200 bp nucleotide fragments to effectively standardise the samples for Illumina NGS platform sequencing. Integrity and quantity was assessed on a gel chip 7500 (Agilent) using a 2100 Bioanalyzer (Agilent Technologies UK Ltd). Concentration of dsDNA was determined using the Quant-iT PicoGreen assay (Invitrogen).

### PCR diagnostics

A HCV PCR diagnostic kit (GeneAmp® EZ rTth RNA PCR, AB life technologies) was used to confirm the presence of the virus, according to the manufacturers instructions, in the HCV infected biopsy samples and the post-transplantation excised HCV infected liver sample used for the enrichment protocol.

### qPCR Assays

Viral nucleotide sequence copy numbers were measured in the amplified material from the various fractions prior to NGS sequencing as well as to estimate the viral genome sequence copy number in the original samples. All liver samples were dissected into equally sized, 2 mm^3^ pieces. Equivalent samples were used for the different extraction procedures and all assays used a Rotorgene 6000 qPCR machine (Rotor-Gene Version 6.1 build 93, 2009, Corbett Research/Qiagen). Data was analysed using Rotor gene software, version 1.7 (Corbett Research/Qiagen).

#### CAV and CPV qPCR

CAV and CPV viral sequence copy number was estimated relative to a standard to determine the genome copy number of CAV and CPV in the liver tissue samples and to acquire a viral nucleic acid copy number estimate from the processed fractions. To produce the standard, amplified fragments of CAV and CPV were individually cloned into pJET1.2 (Fermentas) according to the manufacturer's instructions. Chemically competent *E.coli* cells (One Shot Top10 *E.coli*; Invitrogen) were transformed with this construct and plasmid DNA was extracted from *E.coli* grown overnight in liquid culture (Plasmid mini kit; Qiagen). Plasmid DNA was linearised and quantified using the Quant-iT PicoGreen assay (Invitrogen). A 10-fold dilution series was made by diluting the plasmid DNA in polyinosinic-polycytidylic acid (Poly I:C) from 10^8^ to 1 copy. qPCR was performed on each of the standards, DNA extracted from infected tissue (for genome copy number) as well as the processed DNA and cDNA fractions in triplicate. Amplification was performed using 1 µl of template and 0.3 µM of each primer using the QuantiTect SYBR Green Master Mix (Qiagen) and distilled water to a final volume of 25 µl. After an initial PCR activation step at 95°C for 15 minutes, 45 cycles of amplification were performed consisting of 95°C for 15 seconds, 60°C for 30 seconds and 72°C for 30 seconds.

Hexon gene CAV1 primers

(forward) 5′-TGCTGCCACAATGGTCTTAC-3′ (reverse) 5′- CCACAGTGGGGTTTCTGAAC-3′

NS1 gene CPV2 primers

(forward) 5′- GACTGGGAATCGGAAGTTGA-3′ (reverse) 5′-CAATGCCAGCCTTGATCTTT-3′

#### HCV and HBV qPCR viral genome copy number estimation

These assays are based on the primers and probes previously reported [24]–[25] and modified to fit the QuantiTect Virus qPCR kit (Qiagen) according to the manufacturers instructions. Briefly, the primer final concentrations were at 0.4 µM and the probe final concentrations were at 0.2 µM. Polymerase activation/strand denaturation was at 95°C for 5 minutes, and two step cycling was at 95°C for 15 seconds, and 60°C for 45 seconds for 45 cycles. Total DNA or RNA was extracted from 2 mm^3^ of HBV and HCV liver respectively.

HCV (forward) 5′TGCTAGCCGAGTAGYGTTGG3′

HCV (reverse) 5′ACTCGCAAGCACCCTATCAG3′

HCV (Probe) 5′-[JOE] ACCACAAGGCCTTTCGCGAC [BHQ1] – 3

HBV (forward)5′CAACCTCCAATCACTCACCAAC3′

HBV (reverse) 5′ATATGATAAAACGCCGCAGACAC3′

HBV (Probe) 5′[CY3.5] TCCTCCAATTTGTCCTGGTTATCGCT [BHQ2] 3′

#### qPCR standards and controls

HCV genotype 1a WHO International Standard, NIBSC. 154,881 IU/ml. HBV Eurohep standard reference 1, genotype A, HBsAg subtype adw, WHO International Standard, NIBSC. 1,000,000 IU/ml. Viral nucleic acids were extracted using the High Pure Viral Nucleic Acid Kit, Roche. Viral load was calculated per µl of eluate with no adjustment for kit extraction efficiency. Triplicate dilutions of the HCV and HBV standards were run at neat and 1in 5 serial dilutions together with the samples in triplicate. HCV RNA genome copy number was estimated by using single primers (not pooled) for the reverse transcription step prior to the addition of the second primer and the PCR cycles in order to exclude the –ve strand from the genome copy estimation. The reverse transcription step was at 50°C for 20 minutes.

#### HCV and HBV qPCR viral sequence quantitation in processed sample fractions

HCV and HBV viral sequences in each fraction (RNA or DNA virus enriched, total RNA and total DNA) were quantified against NIBSC controls (HCV 06/100 and HBV 97/750) essentially as described previously with minor modifications [24], [26]. All reactions were carried out in a total volume of 25 µl using Jumpstart Taq readymix (Sigma) and specific forward and reverse primers (HBV1 and HBV2, MAD1 and MAD2 for HCV) were used at a final concentration of 400 nM, and labelled taqman probes at 0.2 µM (BS1 and MAD3 for HBV and HCV respectively). Following a 10 minute denaturation step at 95°C, 50 cycles of 30 seconds at 60°C and 30 seconds at 95°C were performed.

### Illumina NGS protocol

Sequencing was performed using standard Illumina methods. Libraries were created with the Illumina Paired End Genomic DNA Sample Prep kit. Briefly, DNA was sheared into 200–400 bp fragments using a Covaris AFA (Covaris, Woburn, MA), end repaired and an A-overhang added. Illumina paired end adapters were A-T ligated onto the ends of the fragments. Libraries were PCR amplified and each sample sequenced using one lane of an Illumina GA II sequencer generating 76 bp paired end reads. For more detail see Quail *et al* [27].

### Illumina NGS read set trimming

Read set trimming of the NGS data was performed using the CLC Bio Trimming algorithms with the parameters: Failed reads removed on import, ambiguous limit (2), 3′ terminal nucleotides removed (2), homopolymeric tracts of 30 bp removed, SISPA primers removed and minimum number of nucleotides in read allowed (38 nt).

### Illumina NGS Viral Sequence copy number and reference coverage estimation

To assess overall genome coverage and reference nucleotide coverage, the Illumina reads were mapped to complete viral reference genomes (>95% similarity) PVCCP-N for canine parvovirus, AC_000003 for canine adenovirus and EU155829 for hepatitis B virus. Hepatitis C virus reads were first mapped to the 61 reference genomes from the Los Alamos National Laboratory HCV sequence database and the HCV genotype 3 reference sequence NC_009824 with the HCV sub-type 3a consensus genome Ref.3a.DE.HCVCENS1.X76918 used subsequently for best coverage (∼85%) and sample comparison using CLC Bio Genomics Workbench v4 (Katrinebjerg, 8200 Aarhus N, Denmark) and BWA [28]. The references were indexed using bwa index –a IS with bwa aln and bwa sampe used to achieve the paired end alignments. No read mapping was possible to the 5′UTR and the first 400 bp of the core protein sequence for any of the confirmed or unconfirmed HCV geno/subtypes indicative of a novel subtype as defined by this hypervariable region.

### Illumina NGS viral contig de novo assembly

*De novo* assemblies of viral contigs were generated using the CLC Bio Genomics Workbench v4 (Katrinebjerg, 8200 Aarhus N, Denmark) and ABySS 1.2.7 [21] in tandem. For CLC *de novo* assembly both paired and unpaired were co-assembled with a paired read distance min/max at 180/380. For Abyss, a K-mer size of 37 was used, K = 37 and the sequences were assembled as pairs. Contig consensus sequences were mapped to the viral reference genomes to determine total coverage as well as contig size relative to genome size. Sequence alignment to the reference genomes was performed using the sequence alignment algorithms from CLC Genomics workbench v4.0 and bwa. For the CAV and CPV enriched NGS data sets the viral reads were greater than 20% of the total. *De novo* assembly was ineffective probably due to ‘noise’ as a result of accumulated errors in the NGS data sets containing millions of target viral reads. This occurred with both *de novo* assemblers used and was overcome by a combination of splitting the read sets to ≤1 million reads and increasing the alignment stringency of the reduced sets.

## References

[1] Reyes GR, Kim J (1991). Sequence-independent, single-primer amplification (SISPA) of complex DNA populations.. Mol Cell Probes.

[2] Palacios G, Quan P, Jabado OJ, Conlan S, Hirschberg DL (2007). Panmicrobial oligonucleotide array for diagnosis of infectious diseases.. Emerging Inf Dis.

[3] Wang D, Urisman A, Liu YT, Springer M, Ksiazek TG (2003). Viral discovery and sequence recovery using DNA microarrays.. PLoS Biol.

[4] Wang D, Coscoy L, Zylberberg M, Avila PC, Boushey HA, Ganem D (2002). Microarray-based detection and genotyping of viral pathogens.. Proc Natl Acad Sci USA.

[5] Allander T, Emerson SU, Engle RE, Purcell RH, Bukh J (2001). A virus discovery method incorporating DNase treatment and its application to the identification of two bovine parvovirus species.. Proc Natl Acad Sci USA.

[6] Ambrose H, Clewley J (2006). Virus discovery by sequence-independent genome amplification.. Rev Med Virol.

[7] Delwart E (2007). Viral metagenomics.. Rev Med Virol.

[8] Djikeng A, Halpin R, Kuzmickas R, DePasse J, Feldblyum J (2008). Viral genome sequencing by random priming methods.. BMC genomics.

[9] Telenius H, Carter NP, Bebb CE, Nordenskjold M, Ponder BA (1992). Degenerate oligonucleotide-primed PCR: general amplification of target DNA by a single degenerate primer.. Genomics.

[10] Stang A, Korn K, Wildner O, Uberla K (2005). Characterization of virus isolates by particle-associated nucleic acid PCR.. J Clin Microb.

[11] De Souza Luna LK, Baumgarte S, Grywna K, Panning M, Drexler JF (2008). Identification of a contemporary human parechovirus type 1 by VIDISCA and characterisation of its full genome.. Virol J.

[12] De Vries M, Pyrc K, Berkhout R, Vermeulen-Oost W, Dijkman R (2008). Human parechovirus type 1, 3, 4, 5, and 6 detection in picornavirus cultures.. J Clin Microbiol.

[13] De Vries M, Deijs M, Canuti M, van Schaik BD, Faria NR (2011). A sensitive assay for virus discovery in respiratory clinical samples.. PLoS ONE.

[14] Pyrc K, Jebbink MF, Berkhout B, Van der Hoek L (2008). Detection of new viruses by VIDISCA. Virus discovery based on cDNA-amplified fragment length polymorphism.. Meth Mol Biol.

[15] Tan LH, van Doorn R, van der Hoek L, Hien VM, Jebbink MF (2011). Random PCR and ultracentrifugation increases sensitivity and throughput of VIDISCA for screening of pathogens in clinical specimens.. J Inf Dev Count.

[16] Feng H, Taylor JL, Benos PV, Newton R, Waddell K (2007). Human transcriptome subtraction by using short sequence tags to search for tumor viruses in conjunctival carcinoma.. J Virol.

[17] Feng H, Masahiro Shuda, Yuan Chang, Moore PS (2008). Clonal integration of a polyomavirus in human Merkel cell carcinoma.. Science.

[18] Palacios G, Druce J, Du L, Tran T, Birch C (2008). A new arenavirus in a cluster of fatal transplant-associated diseases.. N Engl J Med.

[19] Weber G, Shendure J, Tanenbaum DM, Church GM, Meyerson M (2002). Identification of foreign gene sequences by transcript filtering against the human genome.. Nat Gen.

[20] Victoria J, Kapoor A, Dupuis K, Schnurr DP, Delwart EL (2008). Rapid identification of known and new RNA viruses from animal tissues.. PLoS Path.

[21] Simpson JT, Wong K, Jackman SD, Schein JE, Jones SJ (2009). ABySS: a parallel assembler for short read sequence data.. Genome Res.

[22] Castillo I, Pardo M, Bartolome J, Ortiz-Movilla N, Rodrıguez-Inigo E (2004). Occult hepatitis C infection in patients in whom the etiology of persistently abnormal results of liver-function tests is unknown.. J Inf Dis.

[23] De Marco L, Gillio-Tos A, Fiano V, Ronco G, Krogh V (2009). Occult HCV infection: an unexpected finding in a population unselected for hepatic disease.. PloS1.

[24] Candotti D, Temple J, Owusu-Ofori S, Allain JP (2004). Multiplex real-time quantitative RT-PCR assay for hepatitis B virus, hepatitis C virus, and human immunodeficiency virus type 1.. J Virol Meth.

[25] Hsia CC, Purcell RH, Farshid M, Lachenbruch PA, Yu MW (2006). Quantification of hepatitis B virus genomes and infectivity in human serum samples.. T*ransfusion*.

[26] Weinberger KM, Bauer T, Bohm S, Jilg W (2000). High genetic variability of the group-specific a-determinant of hepatitis B virus surface antigen (HBsAg) and the corresponding fragment of the viral polymerase in chronic virus carriers lacking detectable HBsAg in serum.. J Gen Virol.

[27] Quail MA, Kozarewa I, Smith F, Scally F, Stephens PJ (2008). A large genome center's improvements to the Illumina sequencing system.. Nat Meth.

[28] Li H, Durbin R (2009). Fast and accurate short read alignment with Burrows-Wheeler transform.. Bioinformatics.

